# Changing trends of anaemia prevalence among female medical students in a metropolitan setting: Assessment through self-grading and haematological parameters

**DOI:** 10.12669/pjms.36.7.2793

**Published:** 2020

**Authors:** Arisha Salman, Shamim A. Qureshi, Muhammad Bilal Azmi

**Affiliations:** 1 Dr. Arisha Salman, MBBS. Lecturer, Department of Biochemistry, Dow University of Health Science, Karachi, Pakistan; 2 Dr. Shamim A. Qureshi, Ph.D. Professor, Department of Biochemistry, University of Karachi, Karachi, Pakistan; 3 Dr. Muhammad Bilal Azmi, Ph.D. Assistant Professor, Department of Biochemistry, Dow University of Health Science, Karachi, Pakistan

**Keywords:** Anaemia, Body mass index, Female medical students, Minimum dietary diversity for women

## Abstract

**Objective:**

To determine the trend of anaemia prevalence among female medical students (FMS) through self-grading along haematological parameters and its association with their nutritional indicators.

**Methods:**

This cross-sectional study was conducted at a public sector health university of Karachi among FMS from April to September 2016. After written informed consent, 216 FMS were assessed for height and weight, for nutrition habits by calculating minimum dietary diversity for women (MDD-W) and anaemia status by self-administered validated self-grading anaemia assessment questionnaire (SGAAQ). The haematological parameters were examined in venous blood sample on Sysmex (XN-3000). The data was analysed using IBM SPSS software version 24. Association between anaemia and nutritional indicators was determined by Chi-square and considered significant when p < 0.05.

**Results:**

Anaemia prevalence was 31% with highest frequency among obese (56%) and 29% FMS achieved MDD-W. The mean SGAAQ score, Hb (g/dl), MCV (fl), MCH (pg) and Ret-He (pg) differed significantly (p = <0.001) between anaemic and non-anaemic students. The mean Hb (g/dl) level was significantly higher for FMS who scored MDD-W >5 (p= 0.04).

**Conclusion:**

Malnutrition and anaemia co-exist despite appropriate awareness of anaemia among FMS. It was associated with self-assessment of anaemia and BMI groups but not with dietary diversity in the present study.

## INTRODUCTION

Anaemia is defined as haemoglobin (Hb) below established sex, age, and pregnancy-specific cut-off values in a population influenced by age, smoking and altitude.^1^ The World Health Organization (WHO) estimates anaemia affects 30-55% young adults worldwide.^2^ The National Nutrition Survey (NNS 2018) reported that anaemia affects 41.7% of non-pregnant women in Pakistan. Iron deficiency anaemia remains the number one cause of years lived with disability (YLDs) in Pakistan from 2005 to 2016.3 Sindh has the highest iron deficiency anaemia prevalence of 23.8% (NNS 2018). Jawed, S. et al., reported 33% anaemia frequency in 2017 increasing up to 69% in 2018 among female medical student (FMS), thus emerging as a moderate to major public health problem.^4^

Anaemia has a multifactorial etiology categorized as nutritional, infectious and genetic factors. It is detected by haemoglobin (Hb g/dl) level lower than cut-off value. Reticulocyte Hemoglobin (Ret-He pg) is an early indicator of iron deficiency even before anemia manifestation.^5^ The risk of anaemia is also patterned by level of education, societal practices, socio-economic status especially in women and children.^6^ The general symptoms and signs result from inadequate tissue oxygenation including fatigue, dyspnea on exertion progressing to breathlessness at rest, vertigo, headache and rapid heart-beat and pallor skin, conjunctivae, palms and nail beds. Despite access to better health facilities female medical students (FMS) are at increased risk of anaemia due to their physiological responses to challenging situations, low iron stores, menstrual losses and inadequate food intake.^7^

Malnutrition is claimed to be the most common and preventable cause of anaemia. NNS 2018 shows the double burden of malnutrition (DBM) with almost one in eight adolescent girls being underweight along 11.4% and 5.5% prevalence of overweight and obesity. The assessment and improvement of nutritional indicators like BMI and dietary diversity of FMS can reduce chances of anaemia among them.^8^ This DBM and anaemia challenges the FMS to contribute their fullest potential to the welfare of society and their families. Therefore, aim of the study was to assess the anaemia status through self-grading and its haematological markers and its association with BMI and dietary diversity in FMS.

## METHODS

**Fig.1:**
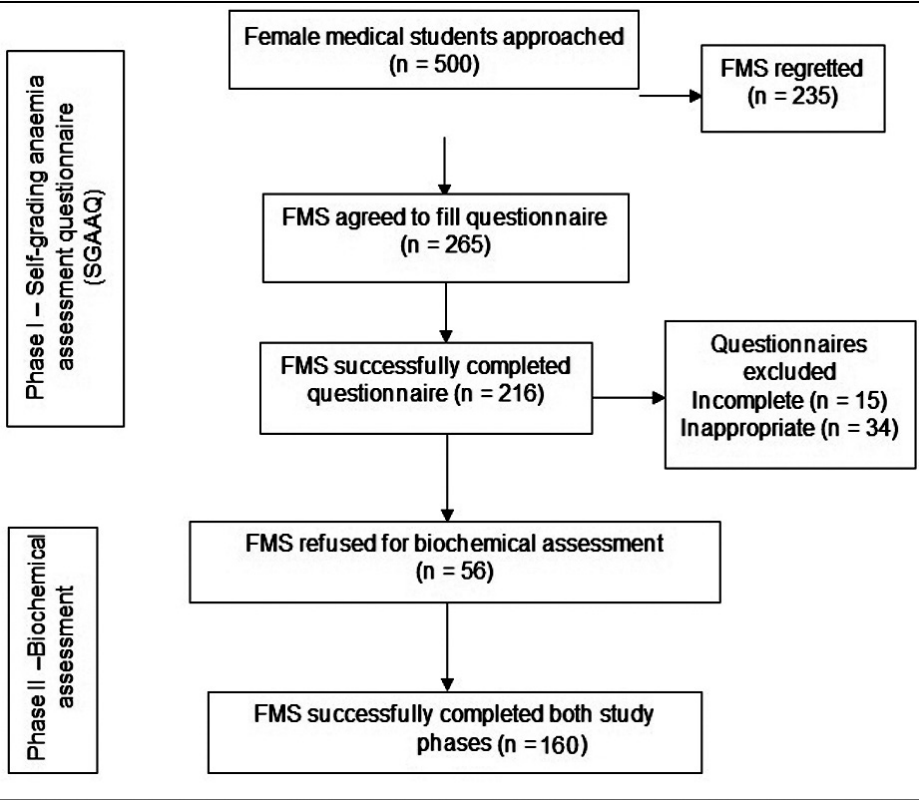
Enrollment of female medical students in study phase I and II.

This is a cross-sectional observational study conducted from April to September 2016 at public sector health university in Karachi. Ethics approval was obtained from Institutional Review Board/ Ethics Review Committee, Dow University of Health Sciences, Karachi via letter No. IRB-659/DUHS/Approval/2016/209. Written informed consent was taken from all participants and briefed about the nature of the study and survey instrument. The sample size was computed using online Rao Soft Sample size calculator by adjusting the margin of error (d) at significance level of 5%, confidence level at 95%, and population size at 20000, with response distribution at 50%. The recommended sample size was 377 subjects. By adding the probability of 35% drop out of subjects, the final sample size was equivalent to 500. The FMS aged 18 to 24 years were enrolled in study by random sampling,100 FMS from each study year in systematic manner. Every third FMS was enrolled after random selection of first one. The enrolment of in study is shown in Fig.1. The participants were excluded from the study who refused to participate, treated for any type of anaemia in past, family history of haemoglobinopathy or blood transfusion in the previous 24 weeks, worm infestation, menstrual disorders, malignancy, known communicable and non-communicable diseases and pregnancy.

### Phase I

The Self-Grading Anaemia Assessment (SGAA) score was calculated by using a structured and pre-tested questionnaire on anaemia status of subjects. At the end of questionnaire total score was calculated. The adequate dietary micronutrient intake of FMS was assessed using “Minimum Dietary Diversity-Women” (MDD-W) score. It is based on the number of food groups consumed prior day or night. Total ten food items divided into three groups were used : Plant-based (green leafy vegetables, fruits and fresh juices), animal based (eggs and poultry, red meat, milk and fish) and staple based (cereals and whole grains). FMS scoring a MDD-W < 5 were labelled as having low dietary diversity while MDD-W > 5–10 as having high dietary diversity.^9^ Height (m) and weight (Kg) were measured to calculate BMI.^10^

### Phase II

Venipuncture was performed according to Standard Operating Procedures (SOPs) and five ml blood sample was collected in ethylenediamine tetra acetic acid (EDTA) containing (purple-toped) tube. Anticoagulated whole blood samples were analyzed for complete blood count within two hours of their collection after calibration and control on automated analyzer, Sysmex (XN-3000). The parameters studied were Haemoglobin (Hb)g/d, Mean cell volume (MCV) fL, Mean Cell Haemoglobin (MCH) pg, Reticulocyte Haemoglobin (Ret-He) pg. The -2SD value of mean Hb level of non-anaemic students with normocytic normochromic red cell morphology was used (13.02 g/dL± 0.62) to establish the reference range for anemia detection. The lower cut -off value was 11.7 g/dL and higher cut-off value was 14.2 g/dL. Peripheral blood film was prepared to study cell morphology within four hours of sample collection and examined initially with /X 10 followed by /X40 under Olympus CX21 microscope. To confirm the quality of peripheral blood smears, the films were examined twice by principal investigator and an expert laboratory technologist. Cronbach’s Alpha was also determined for the consistency among the responses of the study tool. Descriptive data was expressed as mean and standard deviation. The mean values were compared by Mann Whitney U test for two independent groups, Kruskal Wallis test to compare multiple groups as data was not distributed normally. Association between anaemia and nutritional indicators was determined by Chi-square and considered significant when *p <0.05*. Data was analyzed by using Statistical Package for Social Sciences (SPSS) version 24.

## RESULTS

The mean age of the study participants was 19.40 years ± 1.00 (95%CI:19.26 - 19.53) and mean Hb value was 12.1 g/dL ±1.44 (95%CI:11.86 - 12.32). The frequency of anaemic students was 2.3 times more than the non-anaemic FMS. The SGAAQ score and Hb level of FMS was compared at individual, household and community level. The SGAAQ score was significantly different between age groups, study year while birth order for both i.e, SGAAQ score and Hb (g/dl) of FMS (Table-I).

**Table-I:** SGAAQ score and Hb (g/dL) based distribution of significant baseline characteristics among female medical students (n 216).

Characteristics		No. of participants n (%)	SGAAQ score Mean ± S.D n 216	No. of participants n (%)	Hb (g/dL) Mean ± S.D n 160
Age (years)	<19	36 (16.5)	86.94 ± 9.78^a^	23 (14)	12.08 ± 1.15
	19-20	157 (72.3)	81.93 ± 11.94^b^	117 (73)	12.15 ± 1.43
	>20	24 (11)	81.83 ± 13.50^c^	19 (12)	11.71 ± 1.85
	p value		0.03*	p value	0.46
Year of study	First	131(61)	84.80 ± 9.45^a^	89 (55.6)	12.1 ± 1.49
	Other than first year	85 (40)	80.88 ± 12.5^b^	71 (44.4)	11.65 ± 1.43
	p value		0.01*	p value	0.05
Birth order	≤ 2	141(65)	83.97 ± 10.4^a^	97(60.6)	12.4 ± 1.27
	> 2	75 (35)	80.44 ± 11.45^b^	63(39.3)	11.6 ± 1.57^b^
	p value		0.02*	p value	<0.001**

^a^superscript other than ^a^ denotes significantly low value; SGAAQ: Self-Grading Anaemia Assessment Questionnaire, Hb: Haemoglobin, Mann Whitney U test was used to compare two groups. Kruskal Wallis test was used to compare more than two groups.

The mean SGAAQ score was significantly low (*p=0.03*) between non-anaemic and anaemic FMS indicating appropriate awareness of anaemia. The haematological markers of anaemic FMS were below the lower cut-off value except WBC reflecting that adequate anaemia awareness did not improve their anaemia markers (Table-II).

**Table-II:** Self-graded anaemia(SGAAQ) and haematological parameters of female medical students with respect to anaemic status (n = 160)..

Variables		Anaemic Status		p value

		Non-anaemic (n 111)	Anaemic (n 49)	
SGAAQ	SGAAQ score	83.6±12	79.0±13	0.03
Hematological parameters	Hb (g/dL)	12.8±0.6	10.4± 1.0	<0.001
	MCV (fL)	86.0±6.2	76.8±9.9	<0.001
	MCH (pg)	28.0±2.5	23.5±3.9	<0.001
	Ret-He (pg)	29.2± 1.5	28.2±1.9	<0.001
	WBC (10^9^/µL)	8.59±1.8	6.84±2.0	<0.001

All values expressed as mean ± S.D, SGAAQ: Self-Grading Anaemia Assessment Questionnaire, Hb: Haemoglobin, MCV: Mean cell volume, MCH: Mean cell haemoglobin, Ret-He: Reticulocyte Haemoglobin, WBC: White blood cell.

**Fig.2:**
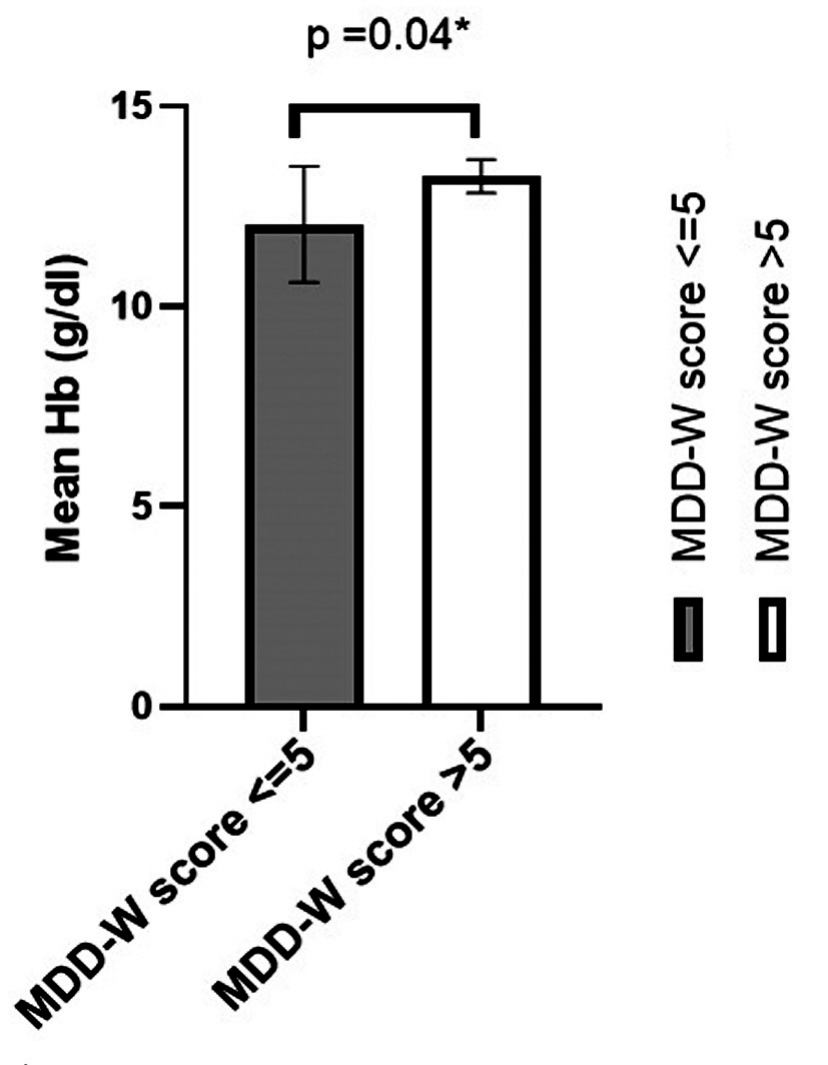
Mean Hb (g/dl) level of Female Medical Students by MDD-W score n=160.

The average BMI of the study participants was 20.31kg/m2 (95%CI:19.85-20.77); underweight (BMI <18.5 kg/m2), overweight (BMI 23–27.5 kg/m2) and obesity (BMI>27.5 kg/m2) were reported in 33.7, 18.5 and 3.7% of the study participants respectively, however the frequency of anaemic overweight/obese FMS was higher than underweight anemic FMS. The significant association of BMI groups of FMS with their anaemia status (p=0.04) was determined but the mean MDD-W score did not differ significantly (Table-III). However, significant low mean Hb (g/dl) was observed for FMS with MDD-W ≤ 5 than those with MDD-W>5(p= 0.04) (Fig.2).

**Table-III:** Distribution of BMI categories of female medical students by their anaemic status and dietary diversity score (n=160).

		Anaemia status			MDD-W Mean ± S.D

		Non anaemic n (%)	Anaemic n (%)	Total n	
BMI ^a^	Normal weight	50 (69)	22 (31)	72	1.90 ± 1.39
	Underweight	45 (79)	12 (21)	57	2.45 ± 1.76
	Overweight	9 (60)	6 (40)	15	1.85 ± 1.83
	Obese	7 (44)	9 (56)	16	2.23 ± 1.85
p value		0.03^b^			0.24^c^

a Asian -specific Body mass index11: Underweight <18.5, Normal weight :18.5 - 22.9, Overweight: 23 - 26.9, Obese ≥27, MDD-W: minimum dietary diversity.

b Chi-square test used to compare proportions,

c Kruskal Wallis test was used to compare more than two groups.

The food groups consumption on previous day by FMS was not associated significantly with their anaemia status. However, the frequency of micronutrient dense plant and staple diet intake was more among non-anaemic FMS than those anaemic but meat and poultry intake was slightly higher among anaemic FMS in contrast to fish intake (Table IV).

**Table-IV:** Association of food groups consumed previous day by female medical students with their anaemic status (n=160).

Food source		Anaemia status			p value

		Non anaemic n (%)	Anaemic n (%)	Total n	
Animal-based	Egg/poultry	41 (49)	42 (50)	83	0.82
	Fish	1 (100)	0 (0)	1	
	Meat	29 (46)	34 (54)	63	
	Milk	48 (52)	45 (48)	93	
Plant-based	Green leafy vegetables	14 (63)	8 (36)	22	0.79
	Fruits	51 (56)	40 (44)	91	
	Fresh juices	20 (56)	16 (44)	36	
Staple-based	Cereals	12 (75)	4 (25)	16
	Whole grains	12 (75)	4 (25)	16

**Fig.3:**
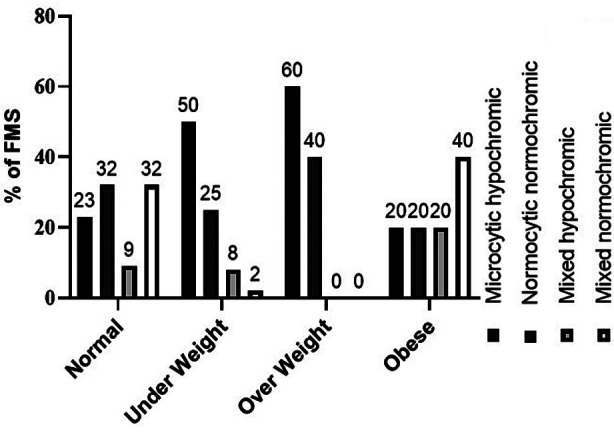
Distribution of morphological types of anaemia by BMI categories of Female Medical Students n 49.

Most of the over-weight anemic FMS presented with microcytic/hypochromic (60%) and normocytic normochromic blood picture (40%) indicating higher frequency of micronutrient deficiency among them (Fig.3).

The significant difference was seen between the means of Mentzer index and anaemia markers (p = <0.01). The Mentzer index <13 (MCV/RBC count) suggests thalassemia trait, and an index of more than 13 suggests iron deficiency.^12^ The four cases were referred for Hb electrophoresis (Table-V).

**Table-V:** Distribution of hypochromic microcytic cases by Menzter index to discriminate iron deficiency anemia and β-thalassemia.

Variable	Hypochromic microcytic anaemia cases n= 24		p value

	Mentzer Index >13 n= 20	Mentzer Index <13 n= 4	
Hb(g/dL)	10.0 ± 1.3	9.48 ± 1.2	ns
RBC count (10^6^/µl)	4.40 ± 0.3	5.17 ± 0.36	<0.01
Hematocrit%	33.87±3.2	32.33 ± 3.3	ns
MCV (fL)	74.80 ± 5.6	62.61 ± 5.9	<0.01
MCH (pg)	22.33 ± 2.5	18.35 ± 2.0	<0.01
Ret-He(pg)	25.8 ± 2.93	15.4 ± 1.97	<0.01
MCV/RBCcount (Menzter index)	17.12 ± 2.1	12.10 ± 0.6	< 0.01

## DISCUSSION

Undernutrition is the most common cause of anaemia in Pakistan which is patterned by various factors like age, gender, race, education, dietary habits and physiological state.^5^,^13^ The female medical students have more understanding and opportunities for anaemia diagnosis and management in context of their educational background and environment.^13^

The FMS self-graded themselves anaemic at individual level factors; age>19 years, study year higher than first year but they were not anaemic biochemically. However, FMS with birth order of greater than two were anaemic biochemically as well. Food insecurity is a significant problem at individual-level among university students.^14^ Present study revealed FMS with birth order of greater than two were anaemic biochemically as well. This was in agreement with Harris-Fry H et al who observed direct relation between the number of family members and food insecurity.^15^ The socioeconomic status was not recognized a significant variable in this study in contrast to recent national survey data.^5^

Present study revealed anemia as a moderate public health issue among FMS; although the FMS had adequate anaemia awareness supported by appropriate self-grading of their anaemia status. This was in agreement with *Sam SM, and Uday Kumar PA*, *2017;* who found inadequate Hb levels among female medical students despite sufficient knowledge of anaemia.^16^

Majority of the FMS were underweight (35%) followed by overweight (11%) and obese (9%) students. The same trend among young university females of Karachi, Pakistan was also observed by Aziz F. *et al*.^8^ Various studies reported higher frequency of anaemia among underweight females in Pakistan.^4^ Surprisingly, anaemia among obese FMS was more frequent in present study supported by negative relation between Hb and BMI in total FMS(data not displayed). It seems that dietary diversity did not affect the anaemia status of obese FMS. Obesity and iron deficiency are intertwined.^2^ Obesity induced inflammation increases hepcidin (iron metabolism regulating protein) concentrations, resulting in reduced iron absorption in intestine or inhibits iron uptake by ferroportin, a cellular iron exporter, from hepatocytes, macrophages and intestinal cells.^17^ It was supported by higher frequency of microcytic hypochromic and normocytic normochromic anaemia among overweight/obese FMS. Dietary diversity was not associated with the anaemic status of FMS as intake of plant and staple diet by non –anaemic FMS was slightly higher than anaemic FMS except that of meat, poultry and eggs. However, the Hb level of FMS with low MDD-W score low reflecting its effect on Hb level. After ruling out Beta thalassemia trait using Mentzer index, low Ret-He in remaining micro/hypo cases indicate iron deficiency. Anemia progressing over a long period may result in IDA and appear as normocytic and normochromic in peripheral circulation. Serum iron and ferritin studies are not indicated in this case with normal Ret-He.^5^

### Limitations of the study

The data was collected at a point in time hence not showing dietary diversity over a year. Biochemical evidence of inflammation (like parasitic infections) was not measured in study sample.

## CONCLUSION

Anemia was prevalent among female university students with higher frequency among obese FMS. It was associated with self-assessment of anaemia and BMI groups but not with dietary diversity. This reflects the co-existence of double burden of malnutrition and anaemia despite knowledge related awareness of anaemia in FMS. This could be possible due to lack of diverse diet intake/knowledge/access and/or obesity.

### Author`s Contribution: AS:

Helped in Data collection, Interpretation of results, manuscript writing and is accountable for the integrity of the data. **SAQ:** Helped in manuscript writing and drafting, reviewed and approved the manuscript. **MBA:** Helped in statistical analysis, interpretation of results, formulation of tables. Reviewed and approved the manuscript.
